# Parasitic infection in the scyphozoan *Rhizostoma pulmo* (Macri, 1778)

**DOI:** 10.1038/s41598-023-31693-7

**Published:** 2023-04-05

**Authors:** Gregorio Motta, Monica Caffara, Maria Letizia Fioravanti, Massimiliano Bottaro, Massimo Avian, Antonio Terlizzi, Perla Tedesco

**Affiliations:** 1 Department of Life Science, University of Trieste, 34127 Trieste, Italy; 2 Department of Integrative Marine Ecology (EMI), Stazione Zoologica Anton Dohrn-Italian National Institute for Marine Biology, Ecology and Biotechnology, 80121 Napoli, Italy; 3 Department of Veterinary Medical Sciences, Alma Mater Studiorum University of Bologna, Ozzano dell’Emilia, 40064 Bologna, Italy; 4 Department of Integrative Marine Ecology (EMI), Genoa Marine Centre (GMC), Stazione Zoologica Anton Dohrn-Italian National Institute of Marine Biology, Ecology and Biotechnology, Villa del Principe, Piazza del Principe 4, 16126 Genoa, Italy

**Keywords:** Ecology, Zoology

## Abstract

Very little information is reported for parasites of cnidarians, therefore, the present work aimed to investigate parasitic infections in one of the most widespread jellyfish in the Mediterranean Sea, *Rhizostoma pulmo.* The goals were to determine prevalence and intensity of parasites in *R. pulmo*, identify the species involved through morphological and molecular analysis, test whether infection parameters differ in different body parts and in relation to jellyfish size. 58 individuals were collected, 100% of them infected with digenean metacercariae. Intensity varied between 18.7 ± 6.7 per individual in 0–2 cm diameter jellyfish up to 505 ± 50.6 in 14 cm ones. Morphological and molecular analyses suggest that the metacercariae belonged to the family Lepocreadiidae and could be possibly assigned to the genus *Clavogalea*. Prevalence values of 100% suggest that *R. pulmo* is an important intermediate host in the life cycle of lepocreadiids in the region. Our findings also support the hypothesis that *R. pulmo* is an important part in the diet of teleost fish, which are reported as definitive hosts of lepocreadiids, since trophic transmission is necessary for these parasites to complete their life cycles. Parasitological data may therefore be useful to investigate fish-jellyfish predation, integrating traditional methods such as gut contents analysis.

## Introduction

In recent times, jellyfish and their blooms have become a discussed topic by marine biologists due to their potential impacts on human activities and ecosystem functioning^1–3^. It is widely recognized that these blooming events always occurred in history, nevertheless, although debates exist concerning changes in frequency and magnitude of jellyfish blooms in relation to climate change and anthropic activities^4,5^, historical data show that jellyfish populations are increasing^6,7^.

Most of jellyfish research focused on their trophic interactions both as predators and preys, while their role as intermediate hosts for a wide variety of parasitic organisms is less known^8–11^. Parasites have been indicated as an important but often neglected component of ecosystems^12^. In this already neglected context, there is paucity of information on parasites of cnidarians: these include Cestoda^13,14^, Trematoda^15,16^, Pycnogonida^17^, other Cnidaria^18^, and Lepadomorpha^19^.

In particular, digenetic trematodes have been shown to use cnidarians as hosts during their life cycle^20–23^. However, the life cycles of most cnidarian-associated species are poorly known^22^. The general life cycle of digeneans is complex, and in most cases is at least partially aquatic, with different developmental stages infecting different hosts: in the definitive host, adults sexually produce eggs that release a miracidium; the miracidium penetrates a first intermediate host and develop into a mother sporocyst, which asexually produces either daughter sporocysts or rediae; further asexual reproduction ultimately produces cercariae that leave the host; in some groups of digeneans, cercariae directly infect the definitive host and ultimately develop into adults; however, a second intermediate host is often involved, where cercariae develop into metacercariae that are trophically transmitted to the definitive host and become adults. To date, very few studies investigated the trophic transmission of metacercariae from jellyfish to fish^24–26^. Equally, few studies addressed the inter-annual presence of digeneans in jellyfish hosts^24,27^. Moreover, recent research mainly focused on hydromedusae and ctenophores as hosts, while data on Scyphozoa are lacking^27^. Up to now, most of the data are spatially limited to South America, Japan and Australia^27^. In the Mediterranean Sea, few zoological studies are available^27–29^, which focused exclusively on the morphological description of trematode species. The knowledge of Scyphozoa–parasite relationships is crucial to determine the role of gelatinous zooplankton as transmitter of parasites to the fish compartment and, in addition, parasitological data could shed light on fish–jellyfish feeding interactions^27^.

This study is aimed at investigating the occurrence of digenean parasites in one of the most complex and widespread jellyfish species in the Mediterranean Sea, namely the barrel jellyfish *Rhizostoma pulmo* (Macri, 1778)^30^. The species is endemic to the Mediterranean Sea and among the biggest jellyfish of this basin (over 40 cm in diameter)^31^. In recent years, *R. pulmo* has been recorded blooming all over the Mediterranean Sea from the Northern to Southern Adriatic Sea, the Ionian Sea, the Eastern and Western Mediterranean, and the Black Sea^32^. In the Gulf of Trieste (North Adriatic Sea), it is present year-round with the highest densities observed from late summer to winter^33^.

Particularly, our goals were to: (1) determine prevalence (P) and intensity (I) of infection of metacercariae in *R. pulmo*; (2) identify the parasite species involved through morphological and molecular analysis, (3) test whether infection parameters change in different body parts and in relation to jellyfish size. To the authors knowledge, this work is the first attempt to study these parasites dynamics in Mediterranean Scyphozoa.

## Results

### Parasite count

*Rhizostoma pulmo* umbrella diameter ranged from 1 to 14.8 cm (5.82 ± 4.18 cm).

All 58 specimens analyzed were parasitized by trematode metacercariae (prevalence 100%). Intensity values ranged between 10 (recorded in a 1 cm juvenile *R. pulmo*) and 556 (recorded in a 13.2 cm specimen), as reported in Table 1.

**Table 1:** Comparison of mean, standard deviation, min and max intensity for each size class interval of *R. pulmo* from the Gulf of Trieste.

Diameter (cm)	No. samples	Mean intensity	Sd intensity	Min intensity	Max intensity
1–2	10	18.7	6.68	10	30
2–4	17	92.2	61	12	200
4–6	7	238	86.2	128	400
6–8	8	311	55.6	243	377
8–10	4	316	26.2	292	351
10–12	3	418	55.1	350	470
12–14	6	505	50.6	443	556
14–16	3	411	17.2	399	431

Class X–Y include X cm up to Y—0.01 cm jellyfish.

**Figure 1:**
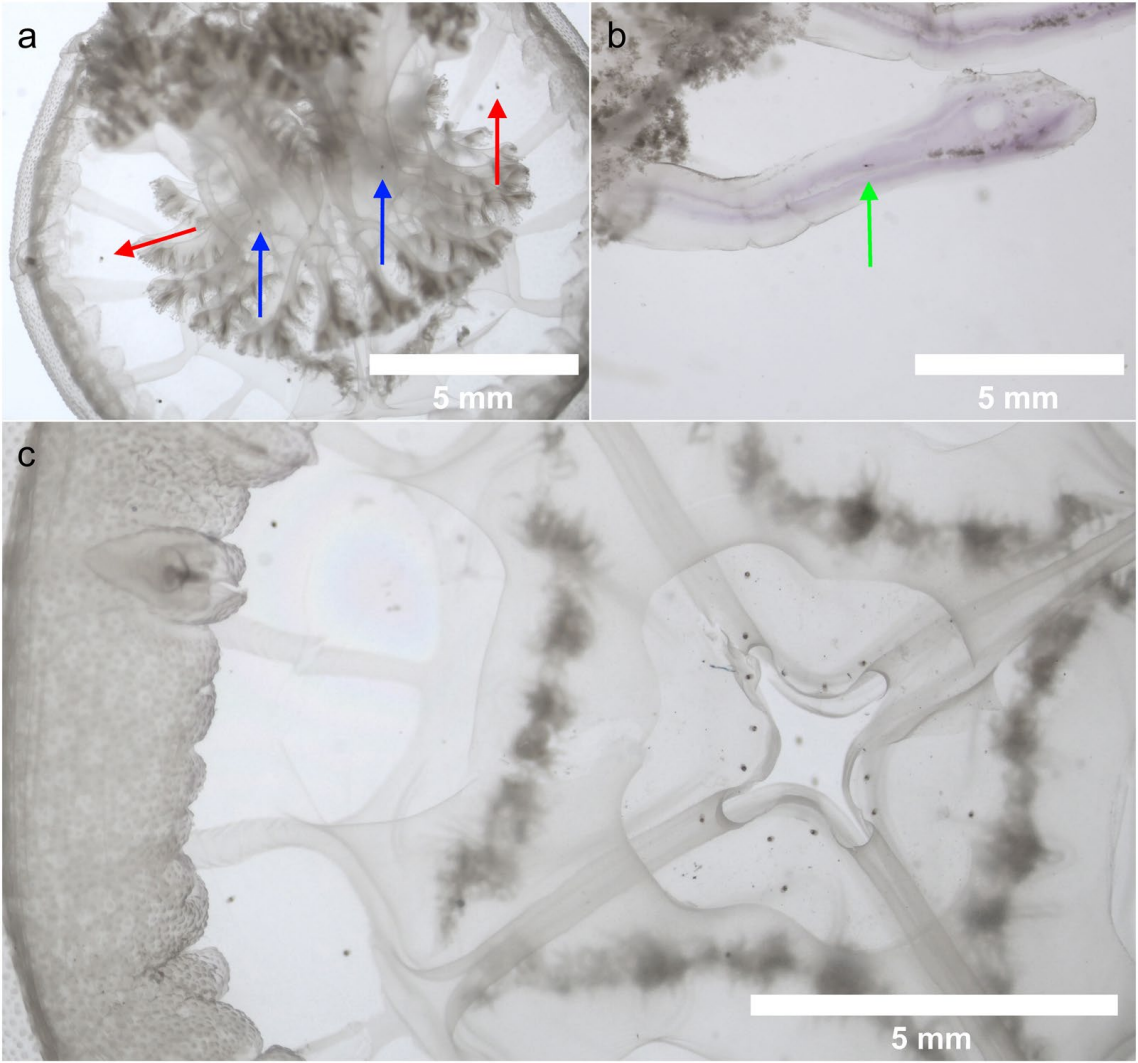
Lepocreadiid metacercariae in different body regions of *R. pulmo*: (**a**) whole jellyfish. Umbrella (red arrow), manubrium (blue arrow), (**b**) oral arm (green arrow) terminal club, (**c**) subumbrellar view, manubrium excised.

Lower intensities were found in the smallest individuals (18.7 ± 6.68) and the number of parasites increased with size up to 505 ± 50.6 in 12–14 cm class size. Considering all the samples, 12,451 parasites have been counted, 6144 in the umbrella and 6307 in the whole manubrium (3863 in the scapulae and 2444 in the oral arms) (Fig. 1).

**Figure 2:**
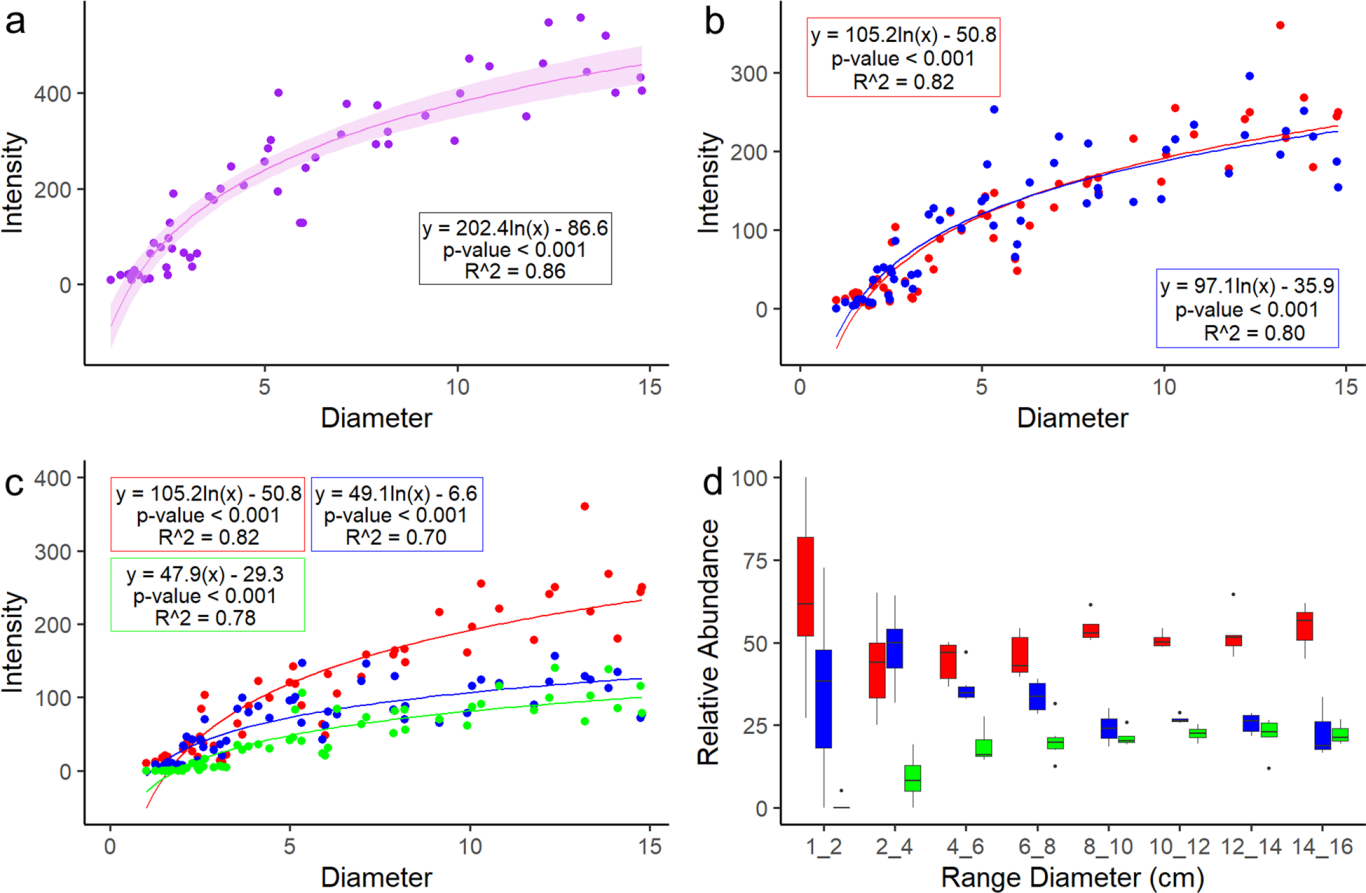
LM for size vs intensity (**a**) in whole body, (**b**) umbrella (red) and whole manubrium (blue), (**c**) umbrella (red), scapulae (blue) and oral arms (green). Coefficients, goodness of fit R2 and P values are reported in each relative box. (**d**) Relative abundance of parasites in umbrella (red), scapulae (blue) and oral arms (green) at different body class size intervals (cm).

Results of the LM provided positive and significant logarithmic regression relationships between the size and the number of parasites for both the whole body and the individual body parts (Fig. 2a–c). Formulas, coefficients, goodness of fit R2 and P values are reported in Fig. 2 and Supplementary Table S1.

In juveniles (1–2 cm), high variability of infection was observed. The most infected body region was the umbrella, followed by the manubrium (Fig. 2d). No parasites were found in the oral arms. In 2 to 4 cm individuals the umbrella and scapulae were equally parasitized, and parasites start to occur in the oral arms at low densities (8.66 ± 5.37%). In jellyfish larger than 4 cm the oral arms become increasingly infected with increasing size of the host (up to 22.4 ± 3.81%), while the scapular area become less infected (23 ± 9.1%). The relative abundance of parasites in the umbrella remains constant (slightly above 50%) irrespective of host size.

### Parasite identification

**Figure 3:**
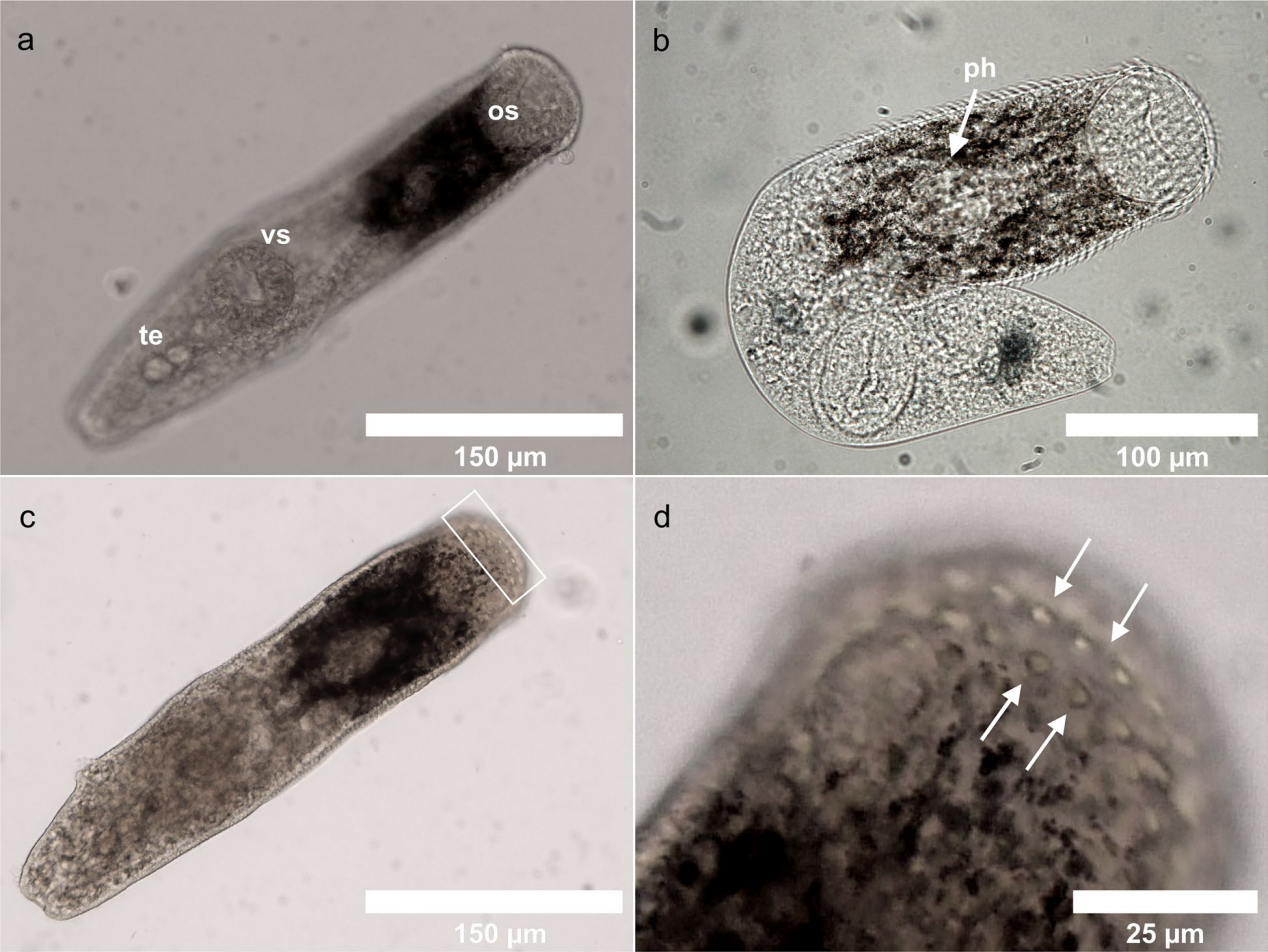
Lepocreadiid metacercariae from *R. pulmo*, light microscopy: (**a**) ventral view, (**b**) cleared specimen, (**c**) dorsal view, square box indicates the double rows of oral spines, (**d**) detail of anterior region showing double rows of oral spines characteristic of the genus *Clavogalea*. *os* oral sucker, *vs* ventral sucker, *te* testes, *ph* pharynx.

**Figure 4:**
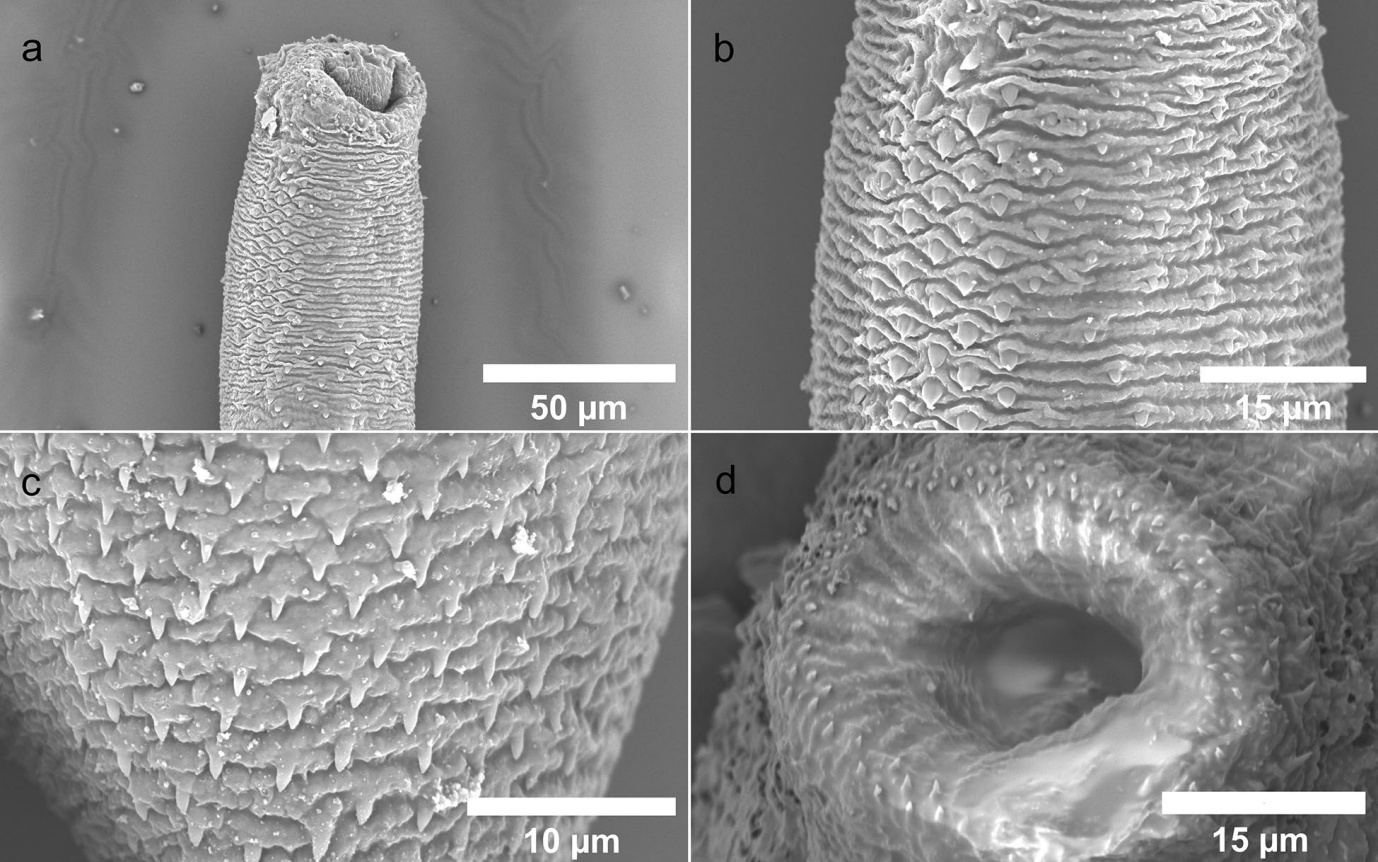
Lepocreadiid metacercariae from *R. pulmo*, SEM micrographs: (**a**) anterior end, (**b**) detail of spines in anterior region, (**c**) detail of spines in posterior region, (**d**) detail of ventral sucker.

The morphological analyses allowed to assign all the unencysted metacercariae analyzed to the family Lepocreadiidae; particularly, the presence of enlarged oral spines and body morphology (Figs. 3, 4) would suggest a possible attribution to the genus *Clavogalea* Bray, in Bray and Gibson^34^ based on the examination of adult specimens, and whose larval stages have never been described.

Morphological description (based on 20 specimens), unit of measurement µm. Body elongate 215.43–461.61 (336.58 ± 56.13) long by 54.81–107.63 (81.63 ± 13.64) wide. Eye-spots pigment scattered in region of pharynx. Tegument spinous. Two rows of enlarged spines around aperture of oral sucker, with rounded base and triangular point, rows alternating. Oral sucker terminal, 45.61–71.58 (60.69 ± 8.76) long by 44.78–80.47 (55.99 ± 9.03) wide, infundibuliform. Ventral sucker rounded 40.99–63.04 (51.52 ± 7.79) long by 42.1–69.8 (54.05 ± 7.47) wide, smaller than oral sucker. Pharynx elongate, oval 36.55–40.54 (39.09 ± 1.59) long. Testes two, elongate, oval, oblique in posterior third of body, separated. Anterior testis 7.43–13.34 (10.30 ± 2.11) long by 12.01–18.19 (14.34 ± 2.35) wide, posterior testis 8.21–16.84 (12.35 ± 2.99) long by 13–17.9 (15.87 ± 1.69) wide. Distance between testes 3.57–7.89 (4.91 ± 1.54). Ovary rounded 6.12–10.77 (7.73 ± 1.84) long by 7.51–15.11 (9.97 ± 3) wide, entire, pre-testicular, separated from anterior testis. Excretory pore terminal.

**Figure 5:**
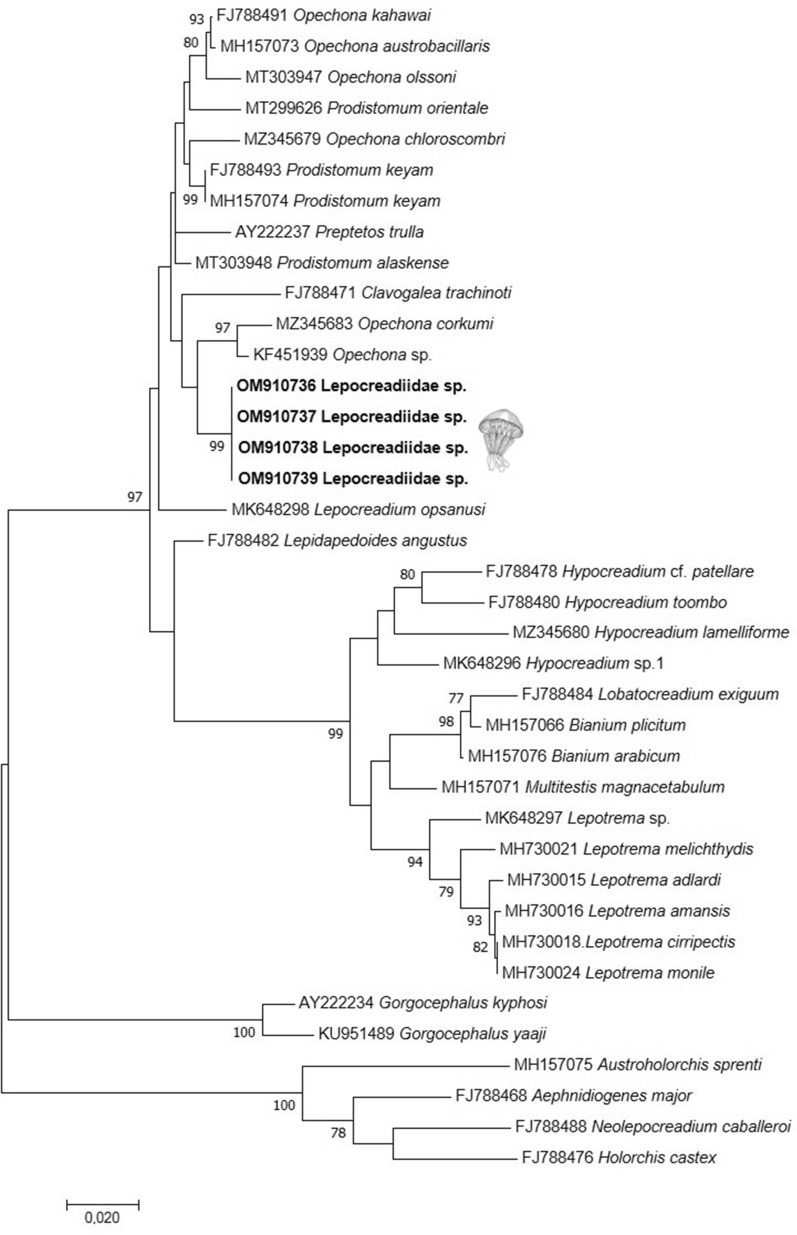
ML tree of lepocreadiid cluster based on 28S rDNA.

Four subjects were successfully amplified and sequenced (28S rDNA), giving a product of 1655 bp. BLAST search gave the highest identity (98%, with a coverage ranging from 99 to 75%) with the lepocreadiid group (*Opechona*, *Preptetos*, *Prodistomum* and *Clavogalea*) with a p-distance ranging from 0.020 to 0.031. The ML tree based on 28S rDNA showed our sequences included in the lepocreadiid cluster (Fig. 5). As already reported in other studies, the interrelationships among the lepocreadiid group which include *Opechona*, *Preptetos*, *Prodistomum* and *Clavogalea* are unresolved and confirmed to be polyphyletic^35,36^.

## Discussion

Data on the occurrence of metacercariae in Scyphozoa are limited^22,27^. To date, as regards the Mediterranean Sea, only few zoological studies aiming only at parasite morphology description are available^27–29^. This work is the first study focused on infection dynamics (in relation to body size and different body regions) in Mediterranean Scyphozoa as well as the first record of jellyfish as second intermediate hosts for digenetic trematodes in the Adriatic Sea.

The high (100%) prevalence found in the present study suggests that *Rhizostoma pulmo* is a key host in the life cycle of lepocreadiid trematodes.

With respect to the fish host, records of lepocreadiid parasites in the Mediterranean Sea include *Opechona bacillaris* (Molin, 1859)^37^, originally described infecting *Centrolophus niger* (Gmelin, 1789)^38^ from Adriatic Sea ^37^,and subsequently reported in *Pomatomus saltatrix* (Linnaeus, 1766)^39^ and *Scomber scombrus* (Linnaeus, 1758)^40^ from different parts of the Mediterranean, Black Sea and Northern Atlantic Sea, and *Prodistomum polonii* (Molin, 1859)^41^ in *Trachurus trachurus* (Linnaeus, 1758)^40^ from the same areas^34,42^. No *Clavogalea* spp. have been reported so far in either intermediate or definitive hosts from the Mediterranean Sea. This genus was erected by Bray^43^; it resembles *Opechona* but differs in having enlarged oral spines and short excretory vesicle ^44^. To date, only two species of *Clavogalea* have been described, namely *Clavogalea gaevskayae* (Bray, 1985)^43^ infecting *Trachinotus botla* (Shaw, 1803)^45^ from South Africa, and *C. trachinoti* (Fischthal & Thomas, 1968)^46^, originally described as *Stephanostomum trachinoti* Fischthal & Thomas, 1968 ^46^ and a senior synonym for *Opechona pseudobacillaris* (Fischthal & Thomas, 1970)^47^ and found from *Trachinotus ovatus* (Linnaeus, 1758)^40^, *T. botla* and *T. coppingeri* (Gunther, 1884)^48^ in Ghana and Australia; of these two species, morphological data are available only from adult stages. Therefore, in the present study it was not possible to confirm the specific identity of our metacercariae based on morphological data. Furthermore, life history and molecular data, which could support a correct species identification, are available only for a fraction of the currently recognized species. Our analysis of 28S rDNA sequences of metacercariae from *R. pulmo* confirm the polyphyletic status among the lepocreadiid group as reported by Bray et al.^35^ and Sokolov et al.^36^. Recent phylogenetic analysis of lepocreadiid based on 28S rDNA^49^ confirmed that interrelationships among members of the family, particularly *Opechona* spp. and other morphologically similar genera (including *Clavogalea*) are unresolved and probably all belong to the genus *Opechona*. Metacercariae collected in the present study were all morphologically similar to each other. Nevertheless, due to possible morphological similarities between metacercariae of different lepocreadiid species and to the limited amount of specimens sequenced, the occurrence of multiple lepocreadiid species in *R. pulmo* cannot be excluded.

In our study, intensity values increase with *R. pulmo* body size. This could be explained by the anatomy and life history of the individuals. Particularly, larger jellyfish may provide an extended contact surface for free-swimming cercariae and may have been exposed for longer time to acquisition and accumulation of parasites, as theorized by Yip^50^. Relative abundances in body regions indicate that infection is not uniform during growth. Most of the studies providing quantitative data on digenean infection in jellyfish focused solely on the umbrella^22^. This work, together with Browne et al.^25^, is one of the first studies that takes in consideration the entire body. Although no differences between the umbrella and the whole manubrium have been found in terms of intensity, we noticed a shift of parasite presence within the scapulae and oral arms during growth. It is interesting to notice that, regardless of the overall intensity in the whole organisms, the relative abundances in the different body regions remain constant in a given size range.

It has been shown experimentally that lepocreadiid cercariae of the genus *Opechona* enter the second intermediate host through active penetration of body tissues^51^. However, our results concerning the relative abundance suggest the idea that parasitization/diffusion in *R. pulmo* by lepocreadiid metacercariae do not take place randomly, but may be influenced by potential drivers, for example the anatomical changes occurring during jellyfish growth. *R. pulmo* preys by active filtration of the water column, umbrella pulsations together with ciliary movement create an inward flux through its central mouth (residual of the mouth of the ephyra stages) in juveniles and, in adults, trough oral arms openings^52,53^. *Rhizostoma* juveniles (1–2 cm diameter) have undeveloped oral arms and they still eat through the central mouth under the umbrella^53,54^. This feeding habit and the related central flux could “pull” swimming cercariae that parasitize the nearest tissues around the stomach pouch and scapulae. Small *R. pulmo* (2–4 cm) begin to possess developed oral arms filled with small mouths. From our observations, during this growth interval the juvenile mouth slowly closes up. These modifications suggest that parasites can now be drawn by both the juvenile mouth (if still open) and oral arms currents. Umbrella and scapulae remain the most parasitized tissues at this stage. Parasites are now found in the oral arms, but low intensity may be due to the fact that oral arms internal circulatory canals are still under development. In fully developed individuals, infection increased in oral arms along with body size. The decrease of intensity observed in the scapulae may be related to its minor role in feeding from this life stage and to oral arms, which are now developed and filled with small canals. Thus, oral arms provide extended external and internal contact surfaces for parasites. At umbrellar level, its wide surface at every stage of jellyfish life, may favor parasite encounter and penetration, thus justifying the almost constant parasite relative abundance.

As mentioned, this is the first study that investigates prevalence and intensity of digenean parasites in *R. pulmo* and takes into consideration all the body in counting parasites in jellyfish from the order Rhizostomeae, to which *R. pulmo* belongs. From our results it is evident how counting parasites only in the umbrella brings to an important underestimation of real intensity, at least in *R. pulmo*. At the same time, this study shows that the distribution of parasites in the organism changes during growth, therefore a whole-body count is the proper way to assess intensity if no solid data about relative abundance in body regions at different sizes is available.

By comparing our data to other studies about jellyfish digeneans, it is evident the difference in prevalence (P) and intensity (I) observed in north Adriatic *R. pulmo*. Morandini et al.^22^ found a P < 10.5 and I < 16 in *Lychnorhiza lucerna* and *Chrysaora lactea* Scyphozoa, Nogueira Junior et al.^27^ found a P < 10 and I < 7 in several hydrozoan species. In Kondo et al.^24^ the mean intensities of the digenean *Lepotrema clavatum* in *Cyanea nozakii*, *Aurelia aurita* and *Chrysaora pacifica* were 219.6, 46.5 and 8.8, respectively. *C. nozakii*, who showed similar intensity^24^ to *R. pulmo*, ranged from 13.3 to 51.0 cm, up to four times the size of the specimens we analyzed.

The higher infection values observed in our study suggest that *R. pulmo* could be a favorable host for this lepocreadiid and, at the same time, the characteristics of Grado Lagoon could provide a favorable spot for digeneans proliferation and transmission. Firstly, the order Rhizostomeae deviates from the anatomy of Scyphozoa. Here, the central mouth is sealed in adults, the manubrium is thick and presents a complex canal system with branched lips forming eight oral arms presenting thousands of mouth openings “mouthlets”^52,55–57^. In *R. pulmo*, the thicker body and the presence of canals that run along all the manubrium up to the umbrella increase the surface of tissue available for infection, if compared to other jellyfish genera as *Aurelia*, *Aequorea*, *Pelagia* where most of the surfaces are limited to the umbrella and gastric pouch^58^.

Marked P and I seasonality of jellyfish digeneans has been observed in temperate environments where water temperature changes drastically throughout the year^24,59^. During winter and spring, only very large individuals (diameter > 30 cm) of *R. pulmo* are available in the Gulf of Trieste. In contrast with small size specimens, characterized by transparent bodies, as *R. pulmo* grows its tissues become thicker and remarkably opaque, thus affecting the samples processing and the accuracy of parasite count, and thus compromising a proper assessment of seasonality.

Parasite presence in the environment is related to the release of free-swimming cercariae from the first intermediate hosts (usually benthic gastropods). No information regarding the first intermediate hosts of lepocreadiid digeneans in the area is available. In literature, digeneans free-swimming cercariae are released from a benthic gastropod in the water column. Køie^51^ first described the presence of *Opechona sp.* cercariae in *Nassarius pygmaeus* from Scandinavia, Barnett et al.^60^ reported the presence of *Stephanostomum*-like cercariae from Australian Nassaridae. Lepocreadiid digenean probably belonging to *Opechona* sp. were identified in gonads and digestive gland of *Buccinanops cochlidium* (Nassaridae) from San Jose´ Gulf, Argentina^59^. In the Gulf of Trieste, two species belonging to the family Nassaridae (superfamily Buccinoidea), *Tritia mutabilis* and *Tritia varicosa* may be plausible candidates^61,62^. Both species inhabits fine sands and fine muddy sands at depths between 2 and 15 m^63^. Grado Lagoon with its sandy bottoms and depths < 10 m provides a wide areal of distribution of these mollusks and the associated parasitic community, thus supporting the I and P measured in *R. pulmo*. At the same time, lagoon shallow waters are likely to increase the chances of encounters among the parasite and the jellyfish.

Focusing on the jellyfish-fish relationship, in the current scenario of climate change where the abundances of digenean parasites and jellyfish may be both favored by warmer waters, along with the recent observations regarding active predation of fish on jellyfish^64^, blooms may favor parasitic infection in the fish compartment. At the current state of the art, few studies focused on predatory interactions between fish and jellyfish that have yet to be fully clarified^9^. To the present day, *R. pulmo* has been demonstrated to be part of the diet of *Trachurus mediterraneus* only, no information of other fish species is available^65,66^. Looking at our results, the 100% of infected individuals suggests that *R. pulmo* is an important intermediate host of lepocreadiid digeneans and therefore plays a key role in their life cycle in the investigated area. This also supports the hypothesis that *R. pulmo* is an important fish prey, since trophic interactions are necessary for these parasites to complete their cycles.

In conclusion, although it was not possible to identify the parasite at species level due to the scarcity of literature information (at both morphological and molecular level) available for these organisms, particularly in the considered area, our work provides important information on the role of *R. pulmo* as intermediate host for lepocreadiids, and useful data as basis for future studies aimed to shed light on the life cycle of these parasites in the Mediterranean Sea. Furthermore, parasitological data may be a useful tool to evaluate trophic interactions between fish and gelatinous organisms (as shown in recent works by Browne et al.^25^ and Duong et al.^26^), another underinvestigated aspect that needs to be fully clarified, integrating traditional methods used in trophic ecology research (gut contents and stable isotopes analysis of fish). Further aspects should be investigated such as the full characterization of these parasites’ life cycle (host species involved, mechanisms, effect on hosts physiology), the effect of seasonality and the possible differences in infection patterns between other Scyphozoan species in the gulf of Trieste.

## Methods

**Figure 6:**
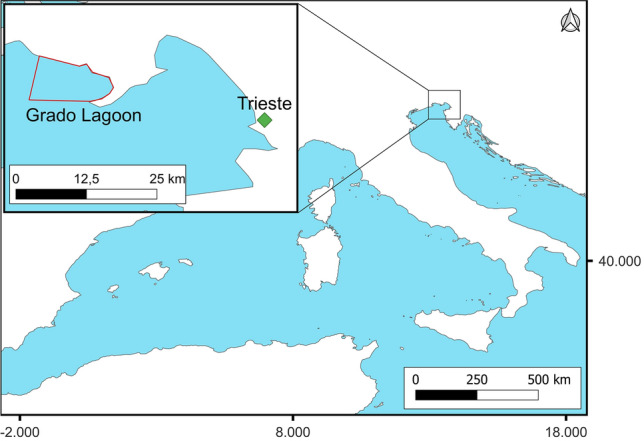
Sampling area of *R. pulmo* (Grado Lagoon, Gulf of Trieste). Map created with QGIS v3.20.3 https://www.qgis.org. Map source from Natural Earth (Free vector and raster map data @ naturalearthdata.com), which material are in the public domain, no permission to use is needed.

*Rhizostoma pulmo* (Macri, 1778) individuals were collected in the Grado Lagoon (Gulf of Trieste, Northern Adriatic Sea, Italy) (Fig. 6) in July/August 2019 and August 2020.

During summer (late July/early August), simultaneous blooms of young to large *R. pulmo* medusae were observed in the Grado Lagoon, therefore, it was possible to collect specimens of different sizes at the same time. All the samples were collected through diving sessions using containers of adequate size to avoid damages to medusae. Samples were brought to the lab and umbrella diameters were measured with a precision caliper and expressed as mean ± standard deviation. A total of 70 jellyfish were collected.

### Parasite count

**Figure 7:**
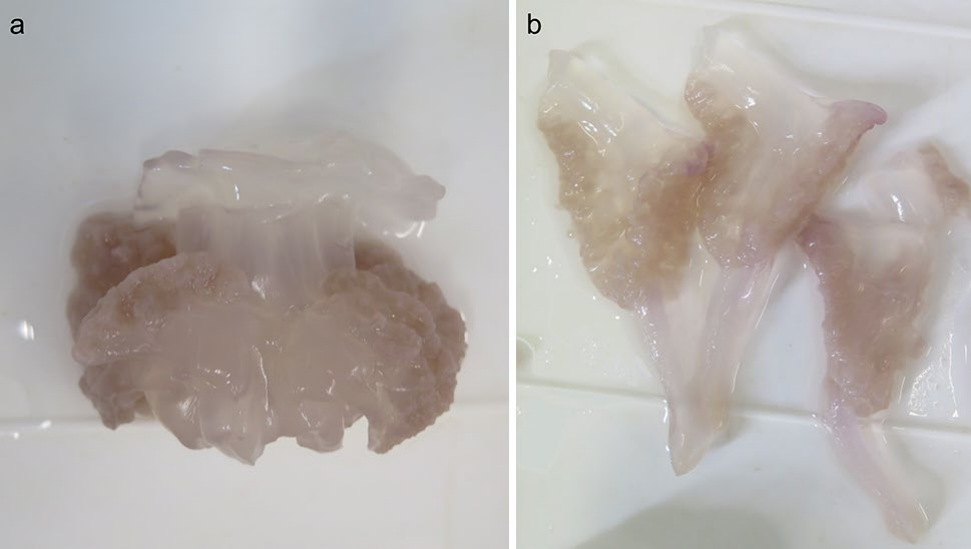
Whole manubrium dissection. Scapular area (**a**) and oral arms (**b**) divided right below scapular level.

Fifty-eight jellyfish (diameter 5.82 ± 4.18 cm) were preserved in 4% formalin-saltwater solution and then stored at room temperature. The umbrella, scapular area (just reported as “scapulae” in the manuscript) and oral arms were separated by a scalpel. Manubrium (scapular area + oral arm) was divided as shown in Fig. 7.

Samples were dissected and the presence of parasites in the different body regions was evaluated under a stereomicroscope (Leica Mz-6). Prevalence (P) and intensity (I) values were defined following Bush et al.^67^.

The relative abundance of parasites in the three different body regions has been calculated as follows:

$$\mathrm{Relative\,Abundance}\left(\mathrm{Body\,region\,x}\right)=\frac{\mathrm{Number\,of\,parasites\,in\,Body\,region\,x}}{\mathrm{Total\,number\,of\,parasites\,in\,the\,sample}}$$

### Parasite identification

For identification, parasites were extracted from fresh tissues of 12 jellyfish and preserved in 70% ethanol for molecular analysis and 10% hot formalin for morphological analysis following Cribb & Bray^68^.

For morphological analysis, 20 metacercariae were subjected to microscopical observation after clarification in Amman’s lactophenol. All measurements were taken with the imaging software NIS-Elements expressed as ranges min–max with mean values ± std.deviation in parentheses. For scanning electron microscopy, fixed specimens were washed three times with phosphate buffer, dehydrated in a graded ethanol series, critical point dried and sputter coated with gold–palladium. Samples were observed using a Phenom XL G2 Desktop SEM (Thermo Fisher Scientific, Eindhoven, The Netherlands) operating at 5 kV.

For molecular analysis, genomic DNA was extracted from four metacercariae by PureLink® Genomic DNA Kit (Life Technologies, Carlsbad, CA, USA), following the manufacturer’s instructions.

The amplification of the D1-D3 region of 28S rDNA was performed with primers U178_f (5′-GCACCCGCTGAAYTTAAG-3′) and L1642_r (5′-CCAGCGCCATCCATTTTCA-3′)^69^, following Gustinelli et al.^70^. The PCR products were electrophoresed on 1% agarose gel stained with SYBR^®^ Safe DNA Gel Stain (Thermo Fisher Scientific, Carlsbad, CA, USA) in 0.5× TBE. All amplicons were purified by NucleoSpin Gel and PCR Cleanup (Mackerey-Nagel, Düren, Germany, CA, USA) and sequenced with an ABI 3730 DNA analyzer (StarSEQ, Mainz, Germany). 28S rDNA amplicons were sequenced with the internal primers 900F (5′-CCGTCTTGAAACACGGACCAAG-3′) and EDC2 (5′-CCTTGGTCCGTGTTTCAAGACGGG-3′) of Lockyer et al.^69^.

The DNA trace files were assembled with ContigExpress (VectorNTI Advance 11 software, Invitrogen, Carlsbad, California) and the consensus sequences were compared with previously published data by BLAST tools (https://blast.ncbi.nlm.nih.gov/Blast.cgi). Multiple sequence alignments (801 positions in the final dataset) of the newly generated sequences together with the all the sequences reported by Curran et al.^49^, were built by BioEdit 7.2.5^71^. Pairwise distance, using a Kimura 2-parameter model and maximum likelihood (ML) tree (BIC = GTR + G, bootstrap of 1000 replicates) were obtained by MEGA version X^72^.

The sequences generated in this study were deposited in GenBank under the accession numbers OM910736-39.

### Statistical analysis

Five Linear Models (LM) were applied to assess the potential relationship between size and intensity in the whole body and in the different body regions. The statistical models were developed under the R statistical environment v.3.6.2^73^. Their specifications were as follows: for each body part (whole body, umbrella, whole manubrium, scapulae and oral arms) the number of parasites has been selected as the response variable and the diameter of jellyfish has been set as the explanatory one. Normality of residuals for each LM was checked with Shapiro–Wilks test and by graphical diagnostics of residuals. In Supplementary Table S1, model formulas, model estimated coefficients and diagnostics were described in detail. Plots were performed using ‘GGplot2’ package^74^. To investigate statistical differences in intensity in the different body parts, two one-way PERMANOVA were performed. In the first one intensity was compared between umbrella and whole manubrium (Supplementary Table S2), in the second intensity was compared between umbrella, scapulae and oral arms (Supplementary Table S3). Euclidean distances were calculated among intensities. The tests were performed using 9999 permutations. When significant, a posteriori pair-wise comparisons were performed among parts via PERMANOVA t statistic with 9999 permutations (Supplementary Table S4). To investigate statistical differences in relative abundance in the different body parts at different body size, a two-way PERMANOVA on the dataset was performed (Supplementary Table S5). The test was performed using 9999 permutations. When significant, a posteriori pair-wise comparisons were performed via PERMANOVA t statistic with 9999 permutations Supplementary (Supplementary Table S6).

Non-parametric analyses were performed using PRIMER-v7^75^ and the add-on package PERMANOVA+^76^.

## Supplementary Information

Supplementary Information.

## Data Availability

All data are contained within the article.
