# Supplementary material for: Parasitic infection in the scyphozoan Rhizostoma pulmo (Macri, 1778)
Source: Sci Rep. 2023 Apr 5;13:5549. doi: 10.1038/s41598-023-31693-7 (PMC10076428; doi:10.1038/s41598-023-31693-7)
Supplement: Supplementary file 1 — Supplementary Information. [file 41598_2023_31693_MOESM1_ESM.pdf]

Supplementary information.

Parasitic infections in mediterranean scyphomedusae: the case of R*hizostoma pulmo* (Macri, 1778) from the Gulf of Trieste

Gregorio Motta [1,2,4], Monica Caffara [3], Maria Letizia Fioravanti [3], Massimiliano Bottaro [2], Massimo Avian [1], Antonio Terlizzi [1,2], Perla Tedesco [3,4,*]

Supplementary Table S1 R summaries for LMs

Model specifications

Model formula (using R notation): the lm function from the stats (version 3.6.2) was used to develop the models.

lm(Intensity in entire body ~ log(Diameter), data = Model)

Residuals:
Min 1Q Median 3Q Max
-146.449 -41.091 5.517 38.241 147.091

Coefficients:
Estimate Std. Error t value Pr(>|t|)
*(Intercept) -86.66 17.95 -4.827 1.11e-05 ****
*log(Diameter) 202.48 10.73 18.863 < 2e-16 ****
---
*Signif. codes: 0 '***' 0.001 '**' 0.01 '*' 0.05 '.' 0.1 ' ' 1*

Residual standard error: 62.33 on 56 degrees of freedom
Multiple R-squared: 0.864, Adjusted R-squared: 0.8616
F-statistic: 355.8 on 1 and 56 DF, p-value: < 2.2e-16

Graphical diagnostic: Distribution of model residuals over the fitted model values: a random distribution is observed indicating no systematic error in model estimated coefficients.

Model specifications

Model formula (using R notation): the lm function from the stats (version 3.6.2) was used to develop the models.

lm(Intensity in entire manubrium ~ log(Diameter), data = Model)

```
Residuals:
    Min      1Q  Median      3Q     Max 
-71.936 -27.283  -0.561  21.281 125.955 

Coefficients:
              Estimate Std. Error t value Pr(>|t|)
(Intercept)    -35.947     10.777  -3.336  0.00152 **
log(Diameter)   97.187      6.443  15.085  < 2e-16 ***
---
Signif. codes:  0 '***' 0.001 '**' 0.01 '*' 0.05 '.' 0.1 ' ' 1

Residual standard error: 37.41 on 56 degrees of freedom
Multiple R-squared:  0.8025, Adjusted R-squared:  0.799 
F-statistic: 227.5 on 1 and 56 DF,  p-value: < 2.2e-16
```

Graphical diagnostic: Distribution of model residuals over the fitted model values: a random distribution is observed indicating no systematic error in model estimated coefficients.

Model specifications

Model formula (using R notation): the lm function from the stats (version 3.6.2) was used to develop the models.

lm(Intensity in umbrella ~ log(Diameter), data = Model)

```
Residuals:
   Min     1Q Median     3Q    Max
-89.48 -23.79  -1.36  21.38 139.15

Coefficients:
              Estimate Std. Error t value Pr(>|t|)
(Intercept)    -50.818     10.751  -4.727 1.58e-05 ***
log(Diameter)  105.288      6.428  16.380  < 2e-16 ***
---
Signif. codes:  0 '***' 0.001 '**' 0.01 '*' 0.05 '.' 0.1 ' ' 1

Residual standard error: 37.33 on 56 degrees of freedom
Multiple R-squared:  0.8273, Adjusted R-squared:  0.8242
F-statistic: 268.3 on 1 and 56 DF,  p-value: < 2.2e-16
```

Graphical diagnostic: Distribution of model residuals over the fitted model values: a random distribution is observed indicating no systematic error in model estimated coefficients.

Model specifications

Model formula (using R notation): the lm function from the stats (version 3.6.2) was used to develop the models.

lm(Intensity in scapulae ~ log(Diameter), data = Model)

```
Residuals:
    Min      1Q  Median      3Q     Max
-53.753 -12.269  -3.779  14.357  71.133

Coefficients:
              Estimate Std. Error t value Pr(>|t|)
(Intercept)     -6.628      7.145  -0.928    0.358
log(Diameter)   49.189      4.271  11.516   <2e-16 ***
---
Signif. codes:  0 '***' 0.001 '**' 0.01 '*' 0.05 '.' 0.1 ' ' 1

Residual standard error: 24.8 on 56 degrees of freedom
Multiple R-squared:  0.7031, Adjusted R-squared:  0.6978
F-statistic: 132.6 on 1 and 56 DF,  p-value: < 2.2e-16
```

Graphical diagnostic: Distribution of model residuals over the fitted model values: a random distribution is observed indicating no systematic error in model estimated coefficients.

Model specifications

Model formula (using R notation): the lm function from the stats (version 3.6.2) was used to develop the models.

lm(Intensity in oral arms ~ log(Diameter), data = Model)

```
Residuals:
    Min      1Q  Median      3Q     Max
-36.521 -12.011  -2.406   8.530  54.822

Coefficients:
              Estimate Std. Error t value Pr(>|t|)
(Intercept)    -29.319      5.492  -5.339 1.76e-06 ***
log(Diameter)   47.998      3.283  14.619  < 2e-16 ***
---
Signif. codes:  0 ‘***’ 0.001 ‘**’ 0.01 ‘*’ 0.05 ‘.’ 0.1 ‘ ’ 1

Residual standard error: 19.07 on 56 degrees of freedom
Multiple R-squared:  0.7924, Adjusted R-squared:  0.7887
F-statistic: 213.7 on 1 and 56 DF,  p-value: < 2.2e-16
```

Graphical diagnostic: Distribution of model residuals over the fitted model values: a random distribution is observed indicating no systematic error in model estimated coefficients.

To investigate statistical differences in intensity in the different body compartments, two one-way PERMANOVA on the dataset was performed (Table S2, S3). Euclidean distances were calculated among intensities. The test was performed using 9999 permutations. When significant, a posteriori pair-wise comparisons were performed among compartments via PERMANOVA t statistic with 9999 permutations (Table S4).

Supplementary Table S2. PERMANOVA: Umbrella vs Whole manubrium

PERMANOVA table of results						
Source	df	SS	MS	Pseudo-F	p-Value	Perms
Comp	1	229.04	229.04	0.030766	0.864	1189
Res	114	8.4871E+05	7444.8			
Total	115	8.4894E+05				

Supplementary Table S3. PERMANOVA: Umbrella vs Scapulae vs Oral arms

PERMANOVA table of results						
Source	df	SS	MS	Pseudo-F	p-Value	Perms
Comp	2	1.20E+05	60076	15.42	0.0001	9663
Res	171	6.65E+05	3894.2			
Total	173	7.86E+05				

Supplementary Table S4. PERMANOVA: Umbrella vs Scapulae vs Oral arms Pairwise Comparison

Pair-Wise Tests			
Groups	t	p-Value	Perms
Umbrella, Scapulae	3.00	0.0029	1030
Umbrella, Oral arms	4.94	0.0001	1081
Scapulae, Oral arms	3.04	0.0036	679

To investigate statistical differences in relative abundance in the different body compartments at different body size, a two-way PERMANOVA on the dataset was performed (Table S5). Euclidean distances were calculated. The test was performed using 9999 permutations. When significant, a posteriori pair-wise comparisons were performed via PERMANOVA t statistic with 9999 permutations (Table S6).

Supplementary Table S5 PERMANOVA: Relative abundance - RangeDiam x Body compartment

PERMANOVA table of results

Source	df	SS	MS	Pseudo-F	p-Value	Perms
RangeDiam	7	0.26289	0.037555	0.00039378	1	9945
Comp	2	25719	12860	134.84	0.0001	9962
RaxCo	14	12108	864.84	9.0682	0.0001	9915
Res	150	14306	95.37			
Total	173	65274				

Supplementary Table S6 PERMANOVA: Relative abundance - RangeDiam x Body compartment. Pairwise Comparison

Resemblance worksheet

Number of permutations: 9999

Factors

Name Levels

Range Diameter (cm) – Body Compartment 24

Groups	t	P(perm)	Unique perms
1-2 Umbrella, 2-4 Umbrella	3.5165	0.0022	9797
1-2 Umbrella, 4-6 Umbrella	2.6407	0.0182	9313
1-2 Umbrella, 6-8 Umbrella	2.0255	0.062	5647
1-2 Umbrella, 8-10 Umbrella	0.90266	0.3895	1000
1-2 Umbrella, 10-12 Umbrella	1.2382	0.24	1000
1-2 Umbrella, 12-14 Umbrella	1.1737	0.2669	2887
1-2 Umbrella, 14-16 Umbrella	0.76615	0.4399	286
1-2 Umbrella, 1-2 Scapulae	3.0825	0.0072	4208
1-2 Umbrella, 2-4 Scapulae	2.6734	0.0133	9820
1-2 Umbrella, 4-6 Scapulae	3.8096	0.0016	9313
1-2 Umbrella, 6-8 Scapulae	3.3555	0.0075	5666
1-2 Umbrella, 8-10 Scapulae	3.5576	0.0056	999
1-2 Umbrella, 10-12 Scapulae	3.353	0.0089	991
1-2 Umbrella, 12-14 Scapulae	3.8786	0.0029	2892
1-2 Umbrella, 14-16 Scapulae	3.1129	0.0151	285
1-2 Umbrella, 1-2 Oral arms	9.1669	0.0001	981
1-2 Umbrella, 2-4 Oral arms	10.099	0.0001	9784
1-2 Umbrella, 4-6 Oral arms	6.0837	0.0001	9304
1-2 Umbrella, 6-8 Oral arms	4.7326	0.0007	5683
1-2 Umbrella, 8-10 Oral arms	3.8209	0.0029	1000
1-2 Umbrella, 10-12 Oral arms	3.7275	0.003	998
1-2 Umbrella, 12-14 Oral arms	4.2077	0.0021	2872

1-2 Umbrella, 14-16 Oral arms	3.2074	0.0098	284
2-4 Umbrella, 4-6 Umbrella	0.53592	0.6032	9823
2-4 Umbrella, 6-8 Umbrella	0.65908	0.5193	9321
2-4 Umbrella, 8-10 Umbrella	2.1307	0.0459	4774
2-4 Umbrella, 10-12 Umbrella	1.4726	0.1455	4733
2-4 Umbrella, 12-14 Umbrella	1.9538	0.0699	8116
2-4 Umbrella, 14-16 Umbrella	1.8123	0.0842	1122
2-4 Umbrella, 1-2 Scapulae	1.2999	0.2144	9762
2-4 Umbrella, 2-4 Scapulae	1.7356	0.091	9840
2-4 Umbrella, 4-6 Scapulae	1.7624	0.0891	9825
2-4 Umbrella, 6-8 Scapulae	1.9851	0.0611	9338
2-4 Umbrella, 8-10 Scapulae	3.3534	0.0026	4674
2-4 Umbrella, 10-12 Scapulae	2.9317	0.0089	4747
2-4 Umbrella, 12-14 Scapulae	3.5111	0.0024	8136
2-4 Umbrella, 14-16 Scapulae	2.9973	0.009	1122
2-4 Umbrella, 1-2 Oral arms	12.36	0.0001	9292
2-4 Umbrella, 2-4 Oral arms	11.78	0.0001	9834
2-4 Umbrella, 4-6 Oral arms	6.3345	0.0001	9788
2-4 Umbrella, 6-8 Oral arms	4.7829	0.0002	9332
2-4 Umbrella, 8-10 Oral arms	3.9043	0.0009	4741
2-4 Umbrella, 10-12 Oral arms	3.7115	0.0008	4691
2-4 Umbrella, 12-14 Oral arms	4.1785	0.0004	8072
2-4 Umbrella, 14-16 Oral arms	3.2048	0.0045	1123
4-6 Umbrella, 6-8 Umbrella	0.32987	0.7325	4250
4-6 Umbrella, 8-10 Umbrella	3.0484	0.012	715
4-6 Umbrella, 10-12 Umbrella	2.0523	0.0488	714
4-6 Umbrella, 12-14 Umbrella	2.3301	0.0325	1983
4-6 Umbrella, 14-16 Umbrella	2.3606	0.0405	220
4-6 Umbrella, 1-2 Scapulae	1.3574	0.1984	9356
4-6 Umbrella, 2-4 Scapulae	1.1541	0.2465	9828
4-6 Umbrella, 4-6 Scapulae	3.6622	0.0022	7336
4-6 Umbrella, 6-8 Scapulae	4.055	0.0021	4242
4-6 Umbrella, 8-10 Scapulae	6.3992	0.0017	714
4-6 Umbrella, 10-12 Scapulae	6.3215	0.0014	715
4-6 Umbrella, 12-14 Scapulae	7.1857	0.0004	1980
4-6 Umbrella, 14-16 Scapulae	5.0933	0.0055	220
4-6 Umbrella, 1-2 Oral arms	24.391	0.0001	988
4-6 Umbrella, 2-4 Oral arms	16.177	0.0001	9784
4-6 Umbrella, 4-6 Oral arms	10.461	0.0001	7966
4-6 Umbrella, 6-8 Oral arms	7.9305	0.0004	4280
4-6 Umbrella, 8-10 Oral arms	7.8632	0.0014	715
4-6 Umbrella, 10-12 Oral arms	7.6424	0.0018	715
4-6 Umbrella, 12-14 Oral arms	7.3825	0.0005	1970
4-6 Umbrella, 14-16 Oral arms	6.4559	0.006	220
6-8 Umbrella, 8-10 Umbrella	2.2239	0.0768	210
6-8 Umbrella, 10-12 Umbrella	1.3933	0.1878	210
6-8 Umbrella, 12-14 Umbrella	1.6475	0.1336	462
6-8 Umbrella, 14-16 Umbrella	1.7025	0.1591	84
6-8 Umbrella, 1-2 Scapulae	1.2113	0.2428	5645
6-8 Umbrella, 2-4 Scapulae	0.6902	0.4966	9329
6-8 Umbrella, 4-6 Scapulae	3.3804	0.0044	4315

6-8 Umbrella, 6-8 Scapulae	3.6414	0.0019	462
6-8 Umbrella, 8-10 Scapulae	5.4855	0.0049	210
6-8 Umbrella, 10-12 Scapulae	5.5014	0.0051	210
6-8 Umbrella, 12-14 Scapulae	6.2371	0.0017	462
6-8 Umbrella, 14-16 Scapulae	4.2996	0.0139	84
6-8 Umbrella, 1-2 Oral arms	20.834	0.0002	125
6-8 Umbrella, 2-4 Oral arms	13.68	0.0001	9237
6-8 Umbrella, 4-6 Oral arms	8.9434	0.0003	4292
6-8 Umbrella, 6-8 Oral arms	6.7534	0.0016	462
6-8 Umbrella, 8-10 Oral arms	6.735	0.0051	210
6-8 Umbrella, 10-12 Oral arms	6.5689	0.005	210
6-8 Umbrella, 12-14 Oral arms	6.2991	0.0021	462
6-8 Umbrella, 14-16 Oral arms	5.5055	0.0121	84
8-10 Umbrella, 10-12 Umbrella	1.3679	0.256	35
8-10 Umbrella, 12-14 Umbrella	0.42388	0.6378	126
8-10 Umbrella, 14-16 Umbrella	0.010627	1	35
8-10 Umbrella, 1-2 Scapulae	1.777	0.1007	1001
8-10 Umbrella, 2-4 Scapulae	1.2166	0.2471	4150
8-10 Umbrella, 4-6 Scapulae	6.6814	0.0013	715
8-10 Umbrella, 6-8 Scapulae	6.8195	0.0058	210
8-10 Umbrella, 8-10 Scapulae	8.6752	0.0291	35
8-10 Umbrella, 10-12 Scapulae	10.933	0.0273	35
8-10 Umbrella, 12-14 Scapulae	11.07	0.0097	126
8-10 Umbrella, 14-16 Scapulae	5.9428	0.0317	35
8-10 Umbrella, 1-2 Oral arms	32.281	0.0013	31
8-10 Umbrella, 2-4 Oral arms	15.556	0.0002	4191
8-10 Umbrella, 4-6 Oral arms	11.896	0.0019	714
8-10 Umbrella, 6-8 Oral arms	9.0895	0.0042	210
8-10 Umbrella, 8-10 Oral arms	11.449	0.0276	35
8-10 Umbrella, 10-12 Oral arms	11.707	0.0286	35
8-10 Umbrella, 12-14 Oral arms	9.0105	0.0066	126
8-10 Umbrella, 14-16 Oral arms	9.3857	0.0278	35
10-12 Umbrella, 12-14 Umbrella	0.51125	0.7365	126
10-12 Umbrella, 14-16 Umbrella	0.86321	0.4546	35
10-12 Umbrella, 1-2 Scapulae	1.4492	0.1742	1001
10-12 Umbrella, 2-4 Scapulae	0.44586	0.6577	4777
10-12 Umbrella, 4-6 Scapulae	6.0362	0.0009	715
10-12 Umbrella, 6-8 Scapulae	6.6291	0.0047	210
10-12 Umbrella, 8-10 Scapulae	9.4401	0.0277	35
10-12 Umbrella, 10-12 Scapulae	16.439	0.0295	35
10-12 Umbrella, 12-14 Scapulae	13.43	0.0094	126
10-12 Umbrella, 14-16 Scapulae	5.9068	0.0278	35
10-12 Umbrella, 1-2 Oral arms	44.201	0.001	31
10-12 Umbrella, 2-4 Oral arms	15.028	0.0002	4169
10-12 Umbrella, 4-6 Oral arms	11.814	0.0014	711
10-12 Umbrella, 6-8 Oral arms	8.997	0.0044	210
10-12 Umbrella, 8-10 Oral arms	14.557	0.0308	35
10-12 Umbrella, 10-12 Oral arms	15.85	0.0297	35
10-12 Umbrella, 12-14 Oral arms	9.2198	0.0068	126
10-12 Umbrella, 14-16 Oral arms	11.909	0.0299	35
12-14 Umbrella, 14-16 Umbrella	0.329	0.7278	56

12-14 Umbrella, 1-2 Scapulae	1.788	0.0941	2897
12-14 Umbrella, 2-4 Scapulae	0.90314	0.3704	8179
12-14 Umbrella, 4-6 Scapulae	5.3676	0.0005	1988
12-14 Umbrella, 6-8 Scapulae	5.2954	0.0028	462
12-14 Umbrella, 8-10 Scapulae	6.6799	0.0091	126
12-14 Umbrella, 10-12 Scapulae	6.9796	0.0081	126
12-14 Umbrella, 12-14 Scapulae	7.7768	0.0084	126
12-14 Umbrella, 14-16 Scapulae	5.1209	0.0185	56
12-14 Umbrella, 1-2 Oral arms	22.495	0.0002	62
12-14 Umbrella, 2-4 Oral arms	14.94	0.0001	7652
12-14 Umbrella, 4-6 Oral arms	10.377	0.0004	1977
12-14 Umbrella, 6-8 Oral arms	7.9173	0.0018	462
12-14 Umbrella, 8-10 Oral arms	8.0139	0.0087	126
12-14 Umbrella, 10-12 Oral arms	7.9094	0.0064	126
12-14 Umbrella, 12-14 Oral arms	7.4755	0.0081	126
12-14 Umbrella, 14-16 Oral arms	6.5898	0.0165	56
14-16 Umbrella, 1-2 Scapulae	1.5196	0.1594	285
14-16 Umbrella, 2-4 Scapulae	1.0332	0.3173	1138
14-16 Umbrella, 4-6 Scapulae	4.9907	0.0083	220
14-16 Umbrella, 6-8 Scapulae	4.8864	0.0117	84
14-16 Umbrella, 8-10 Scapulae	5.9584	0.0309	35
14-16 Umbrella, 10-12 Scapulae	6.5633	0.0283	35
14-16 Umbrella, 12-14 Scapulae	7.1965	0.0169	56
14-16 Umbrella, 14-16 Scapulae	4.3391	0.1031	10
14-16 Umbrella, 1-2 Oral arms	20.749	0.0032	15
14-16 Umbrella, 2-4 Oral arms	12.584	0.0013	986
14-16 Umbrella, 4-6 Oral arms	9.0568	0.0052	220
14-16 Umbrella, 6-8 Oral arms	6.86	0.0125	84
14-16 Umbrella, 8-10 Oral arms	7.3073	0.0309	35
14-16 Umbrella, 10-12 Oral arms	7.2865	0.0279	35
14-16 Umbrella, 12-14 Oral arms	6.554	0.0187	56
14-16 Umbrella, 14-16 Oral arms	5.925	0.0935	10
1-2 Scapulae, 2-4 Scapulae	2.3498	0.0269	9832
1-2 Scapulae, 4-6 Scapulae	0.19644	0.8481	9301
1-2 Scapulae, 6-8 Scapulae	0.10873	0.9141	5658
1-2 Scapulae, 8-10 Scapulae	0.94532	0.369	1000
1-2 Scapulae, 10-12 Scapulae	0.71464	0.5023	953
1-2 Scapulae, 12-14 Scapulae	0.92525	0.3795	2887
1-2 Scapulae, 14-16 Scapulae	0.88986	0.4053	285
1-2 Scapulae, 1-2 Oral arms	4.9953	0.0001	506
1-2 Scapulae, 2-4 Oral arms	4.7888	0.0002	9792
1-2 Scapulae, 4-6 Oral arms	2.1522	0.043	9308
1-2 Scapulae, 6-8 Oral arms	1.5517	0.1493	5686
1-2 Scapulae, 8-10 Oral arms	1.2014	0.2666	998
1-2 Scapulae, 10-12 Oral arms	1.1024	0.2959	1001
1-2 Scapulae, 12-14 Oral arms	1.2953	0.2254	2864
1-2 Scapulae, 14-16 Oral arms	0.95133	0.3682	286
2-4 Scapulae, 4-6 Scapulae	3.8168	0.0011	9823
2-4 Scapulae, 6-8 Scapulae	3.7658	0.0018	9389
2-4 Scapulae, 8-10 Scapulae	5.0756	0.0004	4812
2-4 Scapulae, 10-12 Scapulae	4.6433	0.0004	4817

2-4 Scapulae, 12-14 Scapulae	5.4387	0.0002	8176
2-4 Scapulae, 14-16 Scapulae	4.4413	0.0023	1137
2-4 Scapulae, 1-2 Oral arms	16.246	0.0001	9354
2-4 Scapulae, 2-4 Oral arms	15.457	0.0001	9835
2-4 Scapulae, 4-6 Oral arms	8.9794	0.0001	9795
2-4 Scapulae, 6-8 Oral arms	6.8865	0.0001	9365
2-4 Scapulae, 8-10 Oral arms	5.7461	0.0004	4833
2-4 Scapulae, 10-12 Oral arms	5.533	0.0004	4825
2-4 Scapulae, 12-14 Oral arms	6.1206	0.0001	8167
2-4 Scapulae, 14-16 Oral arms	4.7705	0.0016	1136
4-6 Scapulae, 6-8 Scapulae	1.0167	0.353	4281
4-6 Scapulae, 8-10 Scapulae	4.3146	0.0014	715
4-6 Scapulae, 10-12 Scapulae	4.0126	0.0015	714
4-6 Scapulae, 12-14 Scapulae	4.7101	0.0007	1984
4-6 Scapulae, 14-16 Scapulae	3.429	0.0189	220
4-6 Scapulae, 1-2 Oral arms	23.558	0.0001	986
4-6 Scapulae, 2-4 Oral arms	13.088	0.0001	9798
4-6 Scapulae, 4-6 Oral arms	7.7259	0.0002	8029
4-6 Scapulae, 6-8 Oral arms	5.6601	0.0004	4308
4-6 Scapulae, 8-10 Oral arms	5.9337	0.0014	715
4-6 Scapulae, 10-12 Oral arms	5.6491	0.0017	714
4-6 Scapulae, 12-14 Oral arms	5.212	0.0005	1986
4-6 Scapulae, 14-16 Oral arms	4.7379	0.0045	220
6-8 Scapulae, 8-10 Scapulae	3.0926	0.0231	210
6-8 Scapulae, 10-12 Scapulae	2.8444	0.0295	210
6-8 Scapulae, 12-14 Scapulae	3.3783	0.0117	460
6-8 Scapulae, 14-16 Scapulae	2.4006	0.0592	84
6-8 Scapulae, 1-2 Oral arms	20.898	0.0002	126
6-8 Scapulae, 2-4 Oral arms	10.118	0.0001	9247
6-8 Scapulae, 4-6 Oral arms	5.7847	0.0007	4288
6-8 Scapulae, 6-8 Oral arms	4.1393	0.0075	462
6-8 Scapulae, 8-10 Oral arms	4.6192	0.0085	210
6-8 Scapulae, 10-12 Oral arms	4.3772	0.0102	210
6-8 Scapulae, 12-14 Oral arms	3.8071	0.0022	462
6-8 Scapulae, 14-16 Oral arms	3.6103	0.0098	84
8-10 Scapulae, 10-12 Scapulae	1.0086	0.3677	35
8-10 Scapulae, 12-14 Scapulae	0.52622	0.6078	126
8-10 Scapulae, 14-16 Scapulae	0.20874	0.8591	35
8-10 Scapulae, 1-2 Oral arms	13.779	0.001	31
8-10 Scapulae, 2-4 Oral arms	5.2325	0.0006	4175
8-10 Scapulae, 4-6 Oral arms	1.7672	0.1085	714
8-10 Scapulae, 6-8 Oral arms	0.96451	0.352	210
8-10 Scapulae, 8-10 Oral arms	0.93863	0.3721	35
8-10 Scapulae, 10-12 Oral arms	0.59411	0.6323	35
8-10 Scapulae, 12-14 Oral arms	0.65115	0.5535	126
8-10 Scapulae, 14-16 Oral arms	0.49125	0.7151	35
10-12 Scapulae, 12-14 Scapulae	0.75271	0.4318	126
10-12 Scapulae, 14-16 Scapulae	0.82972	0.372	35
10-12 Scapulae, 1-2 Oral arms	27.578	0.0016	31
10-12 Scapulae, 2-4 Oral arms	6.563	0.0004	4186
10-12 Scapulae, 4-6 Oral arms	3.0459	0.0209	713

10-12 Scapulae, 6-8 Oral arms	1.9274	0.0923	210
10-12 Scapulae, 8-10 Oral arms	3.142	0.0588	35
10-12 Scapulae, 10-12 Oral arms	2.9843	0.0287	35
10-12 Scapulae, 12-14 Oral arms	1.6737	0.0866	126
10-12 Scapulae, 14-16 Oral arms	2.1475	0.1144	35
12-14 Scapulae, 14-16 Scapulae	0.59171	0.5784	56
12-14 Scapulae, 1-2 Oral arms	21.221	0.0005	62
12-14 Scapulae, 2-4 Oral arms	6.652	0.0001	7714
12-14 Scapulae, 4-6 Oral arms	2.7164	0.0218	1981
12-14 Scapulae, 6-8 Oral arms	1.6386	0.1353	462
12-14 Scapulae, 8-10 Oral arms	2.0508	0.0895	126
12-14 Scapulae, 10-12 Oral arms	1.6481	0.1532	126
12-14 Scapulae, 12-14 Oral arms	1.3047	0.227	126
12-14 Scapulae, 14-16 Oral arms	1.3059	0.199	56
14-16 Scapulae, 1-2 Oral arms	8.1393	0.0034	15
14-16 Scapulae, 2-4 Oral arms	3.8717	0.0037	984
14-16 Scapulae, 4-6 Oral arms	1.0404	0.3381	220
14-16 Scapulae, 6-8 Oral arms	0.49292	0.7297	84
14-16 Scapulae, 8-10 Oral arms	0.34589	0.8332	35
14-16 Scapulae, 10-12 Oral arms	0.118	0.9405	35
14-16 Scapulae, 12-14 Oral arms	0.24596	0.8042	56
14-16 Scapulae, 14-16 Oral arms	0.10489	1	10
1-2 Oral arms, 2-4 Oral arms	4.6254	0.0001	8626
1-2 Oral arms, 4-6 Oral arms	10.857	0.0001	993
1-2 Oral arms, 6-8 Oral arms	9.7133	0.0001	125
1-2 Oral arms, 8-10 Oral arms	16.537	0.0009	31
1-2 Oral arms, 10-12 Oral arms	19.436	0.0009	31
1-2 Oral arms, 12-14 Oral arms	11.073	0.0002	63
1-2 Oral arms, 14-16 Oral arms	15.014	0.0031	15
2-4 Oral arms, 4-6 Oral arms	4.6665	0.0002	9805
2-4 Oral arms, 6-8 Oral arms	4.4488	0.0001	9199
2-4 Oral arms, 8-10 Oral arms	4.4899	0.0003	4194
2-4 Oral arms, 10-12 Oral arms	4.9373	0.0002	4170
2-4 Oral arms, 12-14 Oral arms	4.7084	0.0007	7740
2-4 Oral arms, 14-16 Oral arms	4.2043	0.0011	986
4-6 Oral arms, 6-8 Oral arms	0.58473	0.5772	4309
4-6 Oral arms, 8-10 Oral arms	0.92877	0.3827	707
4-6 Oral arms, 10-12 Oral arms	1.3631	0.1962	713
4-6 Oral arms, 12-14 Oral arms	0.99893	0.3219	1981
4-6 Oral arms, 14-16 Oral arms	1.1296	0.3037	220
6-8 Oral arms, 8-10 Oral arms	0.24922	0.8241	210
6-8 Oral arms, 10-12 Oral arms	0.58697	0.5894	210
6-8 Oral arms, 12-14 Oral arms	0.33855	0.7463	462
6-8 Oral arms, 14-16 Oral arms	0.4772	0.6874	84
8-10 Oral arms, 10-12 Oral arms	0.55439	0.6012	35
8-10 Oral arms, 12-14 Oral arms	0.1195	0.9247	126
8-10 Oral arms, 14-16 Oral arms	0.40624	0.534	35
10-12 Oral arms, 12-14 Oral arms	0.23102	0.8729	126
10-12 Oral arms, 14-16 Oral arms	0.021272	0.9711	35
12-14 Oral arms, 14-16 Oral arms	0.17698	0.9079	56
